# A simple-to-use nomogram to predict long term survival of patients undergoing coronary artery bypass grafting (CABG) using bilateral internal thoracic artery grafting technique

**DOI:** 10.1371/journal.pone.0224310

**Published:** 2019-10-24

**Authors:** Tomer Ziv-Baran, Rephael Mohr, Dmitry Pevni, Yanai Ben-Gal

**Affiliations:** 1 Department of Epidemiology and Preventive Medicine, School of Public Health, Sackler Faculty of Medicine, Tel Aviv University, Tel Aviv, Israel; 2 Department of Cardio-Thoracic Surgery, Tel-Aviv Sourasky Medical Center, and Sackler Faculty of Medicine, Tel Aviv University, Tel Aviv, Israel; Royal Infirmary of Edinburgh, UNITED KINGDOM

## Abstract

**Background:**

Several risk scores have been created to predict long term mortality after coronary artery bypass grafting (CABG). Several studies demonstrated a reduction in long-term mortality following bilateral internal thoracic arteries (BITA) compared to single internal thoracic artery. However, these prediction models usually referred to long term survival as survival of up to 5 years. Moreover, none of these models were built specifically for operation incorporating BITA grafting.

**Methods:**

A historical cohort study of all patients who underwent isolated BITA grafting between 1996 and 2011 at Tel-Aviv Sourasky medical center, a tertiary referral university affiliated medical center with a 24-bed cardio-thoracic surgery department. Study population (N = 2,935) was randomly divided into 2 groups: learning group which was used to build the prediction model and validation group. Cox regression was used to predict death using pre-procedural risk factors (demographic data, patient comorbidities, cardiac characteristics and patient's status). The accuracy (discrimination and calibration) of the prediction model was evaluated.

**Methods and findings:**

The learning (1,468 patients) and validation (1,467 patients) groups had similar preoperative characteristics and similar survival. Older age, diabetes mellitus, chronic obstructive lung disease, congestive heart failure, chronic renal failure, old MI, ejection fraction ≤30%, pre-operative use of intra-aortic balloon, and peripheral vascular disease, were significant predictors of mortality and were used to build the prediction model. The area under the ROC curves for 5, 10, and 15-year survival ranged between 0.742 and 0.762 for the learning group and between 0.766 and 0.770 for the validation group. The prediction model showed good calibration performance in both groups. A nomogram was built in order to introduce a simple-to-use tool for prediction of 5, 10, and 15-year survival.

**Conclusions:**

A simple-to-use validated model can be used for a prediction of 5, 10, and 15-year mortality after CABG using the BITA grafting technique.

## Introduction

Coronary artery bypass grafting (CABG) procedures are the most common open heart procedure performed in most heart surgery centres. The standard of care using the left internal thoracic artery (LITA) as a single arterial graft was outlined in the mid-eighties by Loop et al. who showed better long–term patency of a LITA graft to left anterior descending (LAD) compared to that of a saphenous vein graft (SVG) connected to the LAD [1]. Several studies demonstrated a reduction in long-term mortality following bilateral internal thoracic arteries (BITA) compared to single internal thoracic artery (SITA) [2–6].

Several prediction models for long term survival after CABG have been proposed over the last years [7–15]. However, these models usually referred to long term survival as survival of up to 5 years [8–11, 13, 15], while only a few observed 7 or 8-year survival [7, 14, 12]. To the best of our knowledge, no study has published a model predicting survival beyond this period. Moreover, none of these models were built specifically for BITA grafting.

The main objective of the present study was to identify predictors for long term survival after BITA grafting and to build a simple validated prediction tool.

## Methods

### Study design

Historical cohort study.

### Setting

A 24-bed cardio-thoracic surgery department located at the Tel Aviv Sourasky Medical Center, a university affiliated 1500-bed medical center. The medical center is located in the center of Israel and serves a population of nearly 1 million people. The Tel Aviv Sourasky Medical Center ethics committee approved the study and waived the requirement for informed consent (number 0827-18-TLV).

### Participants

All patients who underwent CABG using two ITAs between January 1996 and December 2011 were included in the study. Patients who underwent concomitant procedures were excluded from the study.

In our department the use of BITA grafting has been the standard of care since 1996 [16], and is employed for all patients unless the patient has single vessel disease, increased risk of sternal wound infection (mostly female patients with combination of diabetes and obesity), or the consensus among senior staff is that the patient is too sick to undergo BITA grafting.

### Variables

Data on patients' characteristics were recorded at the time of admission. Demographic data (age and sex); comorbidities: diabetes mellitus (DM), chronic obstructive lung disease (COPD), chronic renal failure (CRF), peripheral and cerebral-vascular disease (PVD, CVD), and any neurologic dysfunction (ND); cardiac characteristics: congestive heart failure (CHF), old and acute myocardial infarction (MI), unstable angina, severely reduced ejection fraction (EF), previous percutaneous transluminal coronary angioplasty (PCI), 3 or more diseased vessels, and left main disease (LM); patient's status: need of pre-operative intra-aortic balloon (IABP), emergency operation, and critical pre-operative status; and surgical characteristics: repeated operation, 3 or more bypass grafts, sequential grafts, the use of saphenous vein grafts, gastro-epiploic, radial grafts, grafts to the right coronary system and the use of off-pump technique, were collected. Old and acute myocardial infarction (MI) were considered as MI more than 7 days before surgery, and MI within the week before the surgery, respectively. Severely reduced ejection fraction was considered as ejection fraction equal or less than 30%. Critical pre-operative status was defined as the status of a patient who needed ventilation or IABP support or inotropic support, or any combination thereof before surgery. Emergency surgery was a surgery performed within the first week after cardiac catheterization due to the patient's condition. Mortality was defined as all-cause mortality occurring up to 15 years after surgery.

### Data sources

All data was collected from patient files and hospital databases by residents or senior physicians. Late mortality was collected from the Israeli Internal Affairs Office National Registry Database.

### Bias

All residents and senior physicians were instructed by one of the researchers (Mohr R) on the data collection in order to minimize collection bias and all data were recorded into an Excel file.

### Sample size

Sample size was calculated according to log-rank test using significance level of 0.5% and power of 90%. The hazard ratio was set to 1.5 and survival at the end of follow-up was considered as 60%. According to these parameters, 1,046 patients were needed to identify predictors for mortality and to build the prediction model.

### Quantitative variables

Age was divided into 5-year categories in order to simplify the use of the prediction model.

### Statistical methods

Categorical variables were reported as number and percentage. Age was reported as mean and standard deviation (SD). Median and interquartile range of the surgery period was also reported. Length of follow-up was observed using reverse censoring method. Study population was randomly divided into two equal size groups. One group was used to identify predictors for mortality and to build the prediction model (learning group) and the second group was used to validate the model (validation group). The characteristics of the two groups were compared in order to evaluate the randomization. Chi-square test, independent samples T test, and Mann Whitney test were used to compare the characteristics between groups. Log-rank test was used to compare survival between groups.

Multivariable cox regression using backward method (likelihood ratio was used as criteria and p>0.1 was set as threshold value for removal). Demographic data, comorbidities, cardiac characteristics, and patients' status were included in the multivariable cox regression. Nomogram was built in order to introduce a simple tool for prediction of 5, 10, and 15-year survival. We also evaluated the baseline hazard at these time points and reported the coefficients of the variables that were included in the final model.

Calibration plots (predicted vs. observed values) were drawn in order to evaluate the prediction model. A predicted value indicates the predicted survival probability at a single time point, and observed value refers to the corresponding Kaplan-Meier survival estimate.

Harrell's concordance (C) statistic was used to evaluate the overall model discrimination ability. The area under the receiver operating characteristics curve was used to evaluate the discrimination ability of the model at these time points in both the learning and validation groups.

All statistical tests were two sided and p<0.05 was considered as statistically significant. SPSS (IBM SPSS Statistics for Windows, version 24, IBM corp., Armonk, NY, USA, 2016) and R (version 3.5.1, R Foundation for Statistical Computing, Vienna, Austria, 2018) were used for all statistical analysis. The following "R" packages were used: “rms” package (Harrell FE Jr. rms: Regression Modeling Strategies, ver. 5.1–2), “survival” package (Therneau TM. survival:Survival Analysis, ver. 2.42–6), "prodlim" package (Gerds TA. prodlim:Product-Limit Estimation for Censored Event History Analysis, ver. 2018.04.18), "dynpred" package (Putter H. Companion Package to "Dynamic Prediction in Clinical Survival Analysis", ver. 0.1.2), and "survivalROC" package (Heagerty PJ. survivalROC:Time-dependent ROC curve estimation from censored survival data, ver. 1.0.3).

## Results

The study cohort included 2,935 patients who underwent BITA grafting between 1996 and 2011 (median January 2001). The median follow-up time was 15 years (IQR 12–15). One thousand four hundred and sixty eight patients were included in the learning group and 1,467 in the validation group. Comparisons of patients' characteristics are presented in Table 1.

**Table 1:** Comparison of patients' characteristics between the learning and the validation groups.

	Group		
	Learning (n = 1468)	Validation (n = 1467)	p
Demographic characteristics:			
Male	1194 (81.3%)	1214 (82.8%)	0.317
Age (years), mean (SD)	65.2 (10.5)	65.3 (10.6)	0.851
Period, median (IQR)	12/2000	03/2001	0.258
	(03/1998-01/2005)	(04/1998-03/2005)	
Comorbidities:			
DM	495 (33.7%)	506 (34.5%)	0.659
DM EOD	88 (6.0%)	93 (6.3%)	0.698
COPD	77 (5.2%)	68 (4.6%)	0.446
CRF	105 (7.2%)	99 (6.7%)	0.667
PVD	262 (17.8%)	274 (18.7%)	0.561
CVD	142 (9.7%)	134 (9.1%)	0.617
ND	64 (4.4%)	51 (3.5%)	0.218
Cardiac characteristics:			
CHF	305 (20.8%)	308 (21%)	0.884
Old MI	575 (39.2%)	540 (36.8%)	0.188
Acute MI	278 (18.9%)	297 (20.2%)	0.372
UAP	824 (56.1%)	772 (52.6%)	0.057
EF ≤ 30%	109 (7.4%)	85 (5.8%)	0.075
NOVS ≥ 3	1014 (69.1%)	1131 (77.1%)	0.439
LM	479 (32.6%)	457 (31.2%)	0.391
PTCA	231 (15.7%)	246 (16.8%)	0.448
Patient's status:			
IABP	97 (6.6%)	87 (5.9%)	0.449
Critical	104 (7.1%)	97 (6.6%)	0.612
Emergency	208 (14.2%)	234 (16%)	0.177
Surgical characteristics:			
REDO	33 (2.2%)	29 (2%)	0.610
Bypass ≥ 3	1079 (73.5%)	1122 (76.5%)	0.062
Sequential	650 (44.3%)	667 (45.5%)	0.517
SVG	503 (34.3%)	523 (35.7%)	0.431
GEA	179 (12.2%)	174 (11.9%)	0.782
Right system	881 (60%)	925 (63.1%)	0.891
RADIAL	40 (2.7%)	43 (2.9%)	0.736
OPCAB	356 (24.3%)	328 (22.4%)	0.225

DM—Diabetes mellitus; EOD—End organ damage; COPD—Chronic obstructive lung disease; CRF—Chronic renal failure; PVD—Peripheral vascular disease; CVD–Cerebral vascular disease; ND—Neurologic dysfunction; CHF—Congestive heart failure; MI—Myocardial infarction; UAP—Unstable angina pectoris; EF—Ejection fraction; NOVS–Number of vessels; LM—Left main disease; PTCA—Percutaneous transluminal coronary angioplasty; IABP—Intra-aortic balloon; REDO—Repeated operation; SVG—Saphenous vein graft; GEA—gastro-epiploic graft; OPCAB—Off-pump coronary artery bypass

**Fig 1:**
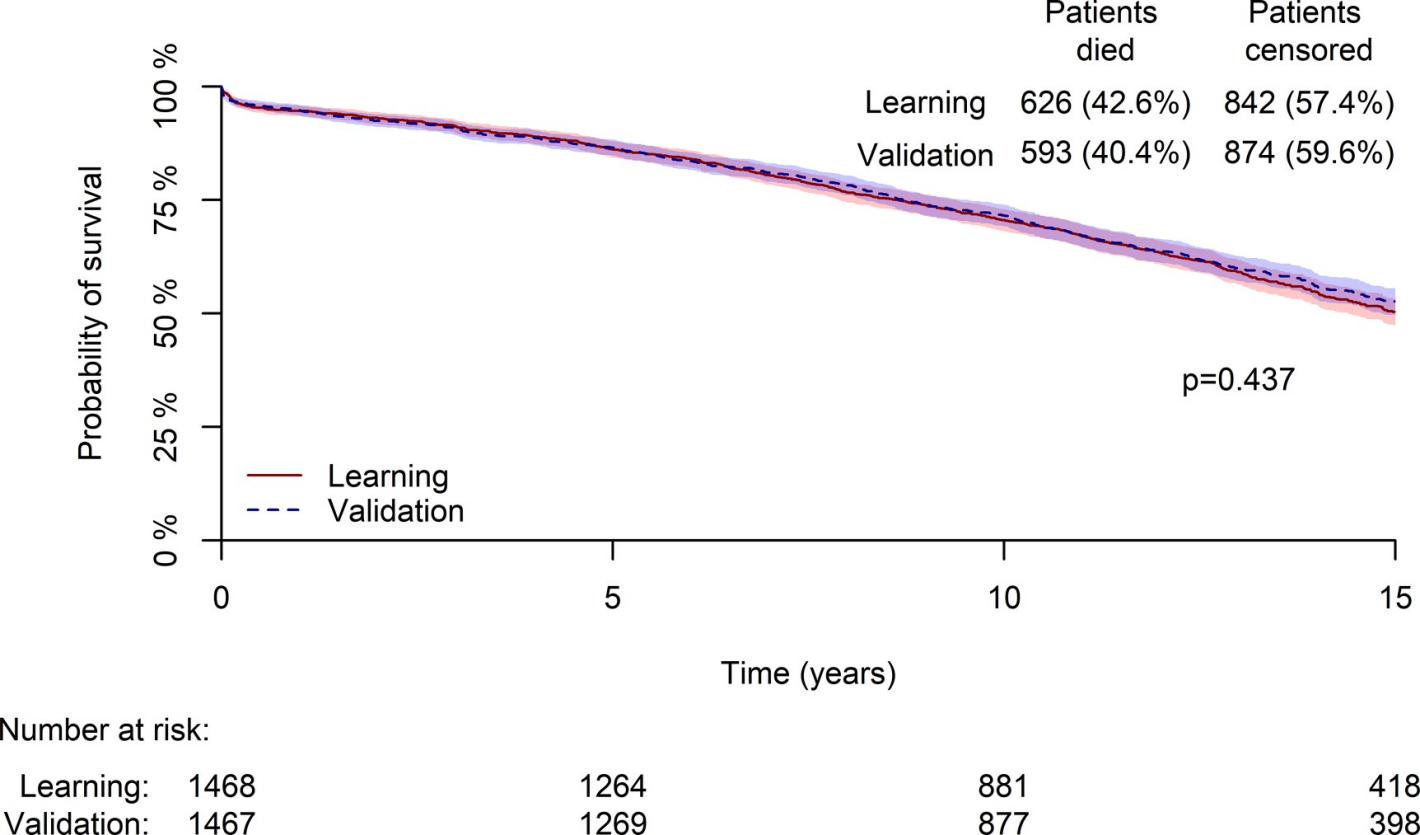
Kaplan-Meir curve demonstrating the cumulative survival during the follow-up period in the learning and validation groups.

Six hundred twenty-six (42.6%) patients in the learning group and 593 (40.4%) patients in the validation group died during the follow-up (p = 0.437). The median survival time was not reached over the 15 years of follow up. The cumulative 5, 10, and 15 year survival rates were 0.861, 0.706, and 0.503, respectively for the learning group, and 0.865, 0.716, and 0.526, respectively, for the validation group (Fig 1).

In multivariable analysis based on the learning group, older age, diabetes mellitus, COPD, CHF, CRF, old MI, EF≤30%, pre-operative intra-aortic balloon, and PVD were significant predictors for mortality in the learning group (Table 2).

**Table 2:** Predictors for mortality in the learning group and the multivariable cox regression coefficients.

Predictor	Coefficient	HR (95%CI)	p
Age (years)			<0.001
<55		1	
55–59	0.5858	1.796 (1.178–2.739)	
60–64	0.7733	2.167 (1.450–3.238)	
65–69	1.1821	3.261 (2.260–4.705)	
70–74	1.3961	4.040 (2.834–5.759)	
75–79	1.8342	6.258 (4.370–8.962)	
80+	2.0434	7.700 (5.216–11.366)	
DM	0.2795	1.323 (1.122–1.559)	0.001
COPD	0.4283	1.534 (1.127–2.089)	0.007
CHF	0.2932	1.341 (1.114–1.614)	0.002
CRF	0.3797	1.460 (1.134–1.880)	0.003
Old MI	0.2225	1.249 (1.061–1.470)	0.007
EF≤30%	0.3600	1.433 (1.102–1.863)	0.007
IABP	0.4476	1.565 (1.169–2.095)	0.003
PVD	0.4424	1.556 (1.298–1.866)	<0.001

The baseline hazards for 5, 10 and 15 years were 0.02689, 0.06678 and 0.14259, respectively.

DM—Diabetes mellitus; COPD—Chronic obstructive lung disease; CHF—Congestive heart failure; CRF—Chronic renal failure; MI—Myocardial infarction; EF—Ejection fraction; IABP—Intra-aortic balloon; PVD—Peripheral vascular disease

**Fig 2:**
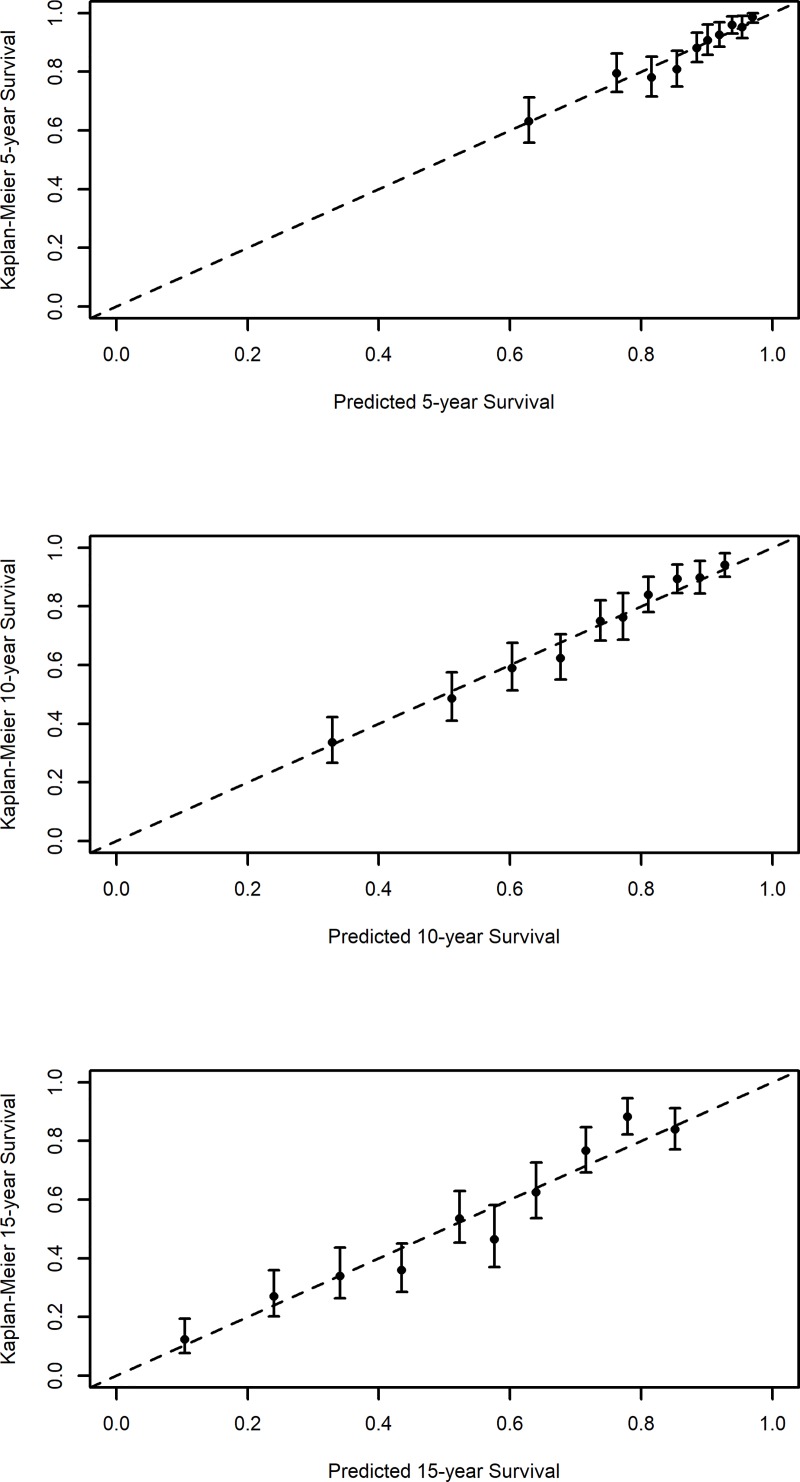
Calibration curve of the multivariable cox regression model based on the learning group.

**Fig 3:**
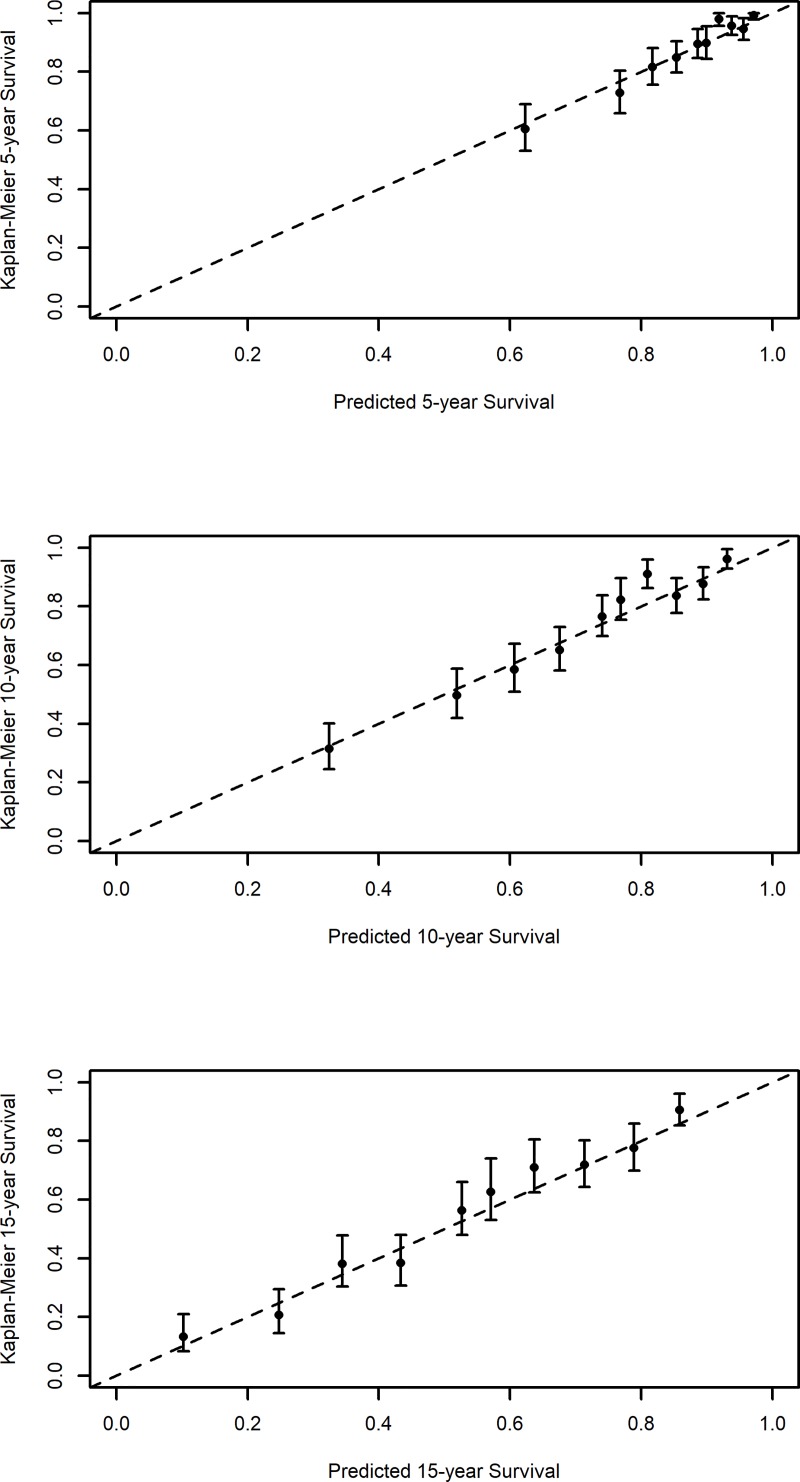
Calibration curve of the prediction model based on the validation group.

The calibration curves of the model presented good calibration at the three time points, in both the learning and validation groups (Fig 2 and Fig 3).

Harrell's C was 71.5% in the learning group. The AUCs of cox model for the learning group for 5, 10, and 15 years were 0.742, 0.755, and 0.762, and for the validation group 0.766, 0.763, and 0.770, respectively. The ROC curves are presented in S1 and S2 Figs.

**Fig 4:**
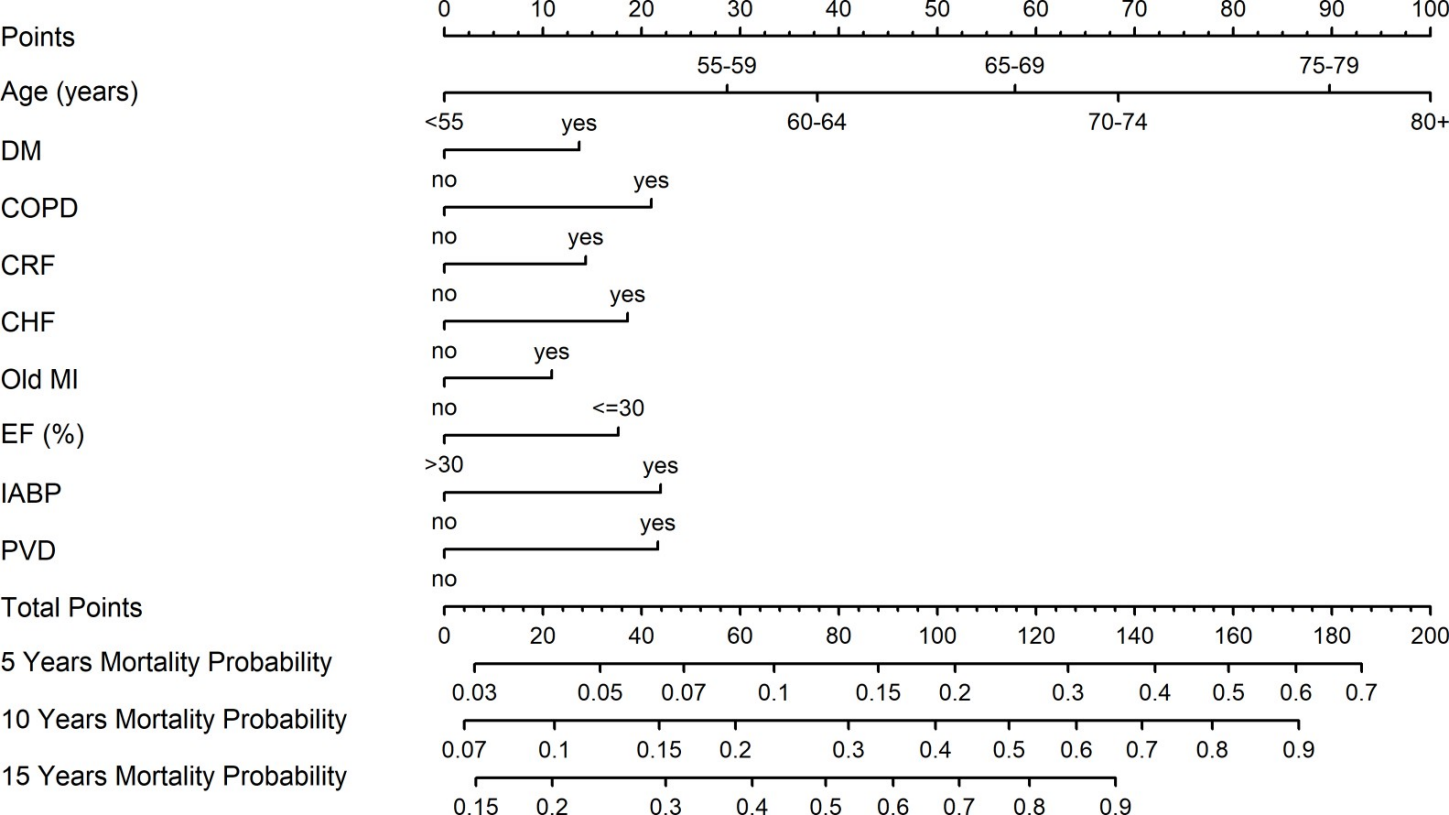
Nomogram presenting the prediction model for 5, 10 and 15-year survival. Nomogram shows cox model for prediction of mortality 5, 10, and 15 years after surgery. Each parameter has corresponding values (points) that appear in the upper toolbar (also in Table 3). Summarized total points should be applied on the bottom scale ("Total Point") to obtain probability of mortality 5, 10 and 15 years after surgery. DM—Diabetes mellitus; COPD—Chronic obstructive lung disease; CHF—Congestive heart failure; CRF—Chronic renal failure; MI—Myocardial infarction; EF—Ejection fraction; IABP—Intra-aortic balloon; PVD—Peripheral vascular disease.

Fig 4 shows a nomogram based on the cox model with prediction of mortality 5, 10, and 15 years after surgery. Table 3 indicates the points for each variable in the nomogram and Table 4 shows the total points for several main probabilities of mortality.

**Table 3:** Points that each variable represents in the nomogram.

Predictor	Points
Age (years)	
<55	0
55–59	29
60–64	38
65–69	58
70–74	68
75–79	90
80+	100
DM	14
COPD	21
CHF	14
CRF	19
Old MI	11
EF≤30%	18
IABP	22
PVD	22

DM—Diabetes mellitus; COPD—Chronic obstructive lung disease; CHF—Congestive heart failure; CRF—Chronic renal failure; MI—Myocardial infarction; EF—Ejection fraction; IABP—Intra-aortic balloon; PVD—Peripheral vascular disease

**Table 4:** Predicted mortality for 5, 10, and 15 years after surgery, according to sum of points on the nomogram.

Probability of mortality	Years after surgery		
	5	10	15
5%	32		
10%	67	22	
15%	88	44	6
20%	104	59	22
25%	116	71	34
30%	127	82	45
35%	136	91	54
40%	144	100	62
45%	152	107	70
50%	159	115	77
55%	166	121	84
60%	173	128	91
65%	179	135	98
70%	186	142	104
75%	193	148	111
80%		156	119
85%		164	127
90%		173	136
95%		186	149

## Discussion

Several risk scores were developed to evaluate the risk associated with early mortality after CABG [17–19], and several risk scores were also developed to evaluate long term mortality [7–15]. However, these models evaluated late mortality of up to 8 years post-operatively. Similar prediction models were also developed in other medical areas, for example: a model to predict mortality in patients with prostate cancer [20], a model to predict pulmonary hypertension based on findings of CT pulmonary angiography [21], and a model to predict early relapse in patients with Crohn`s disease [22].

Several studies have shown significant benefit of BITA grafting compared to SITA grafting [16, 23–26]. Therefore, models that were built according to patients who mostly underwent SITA grafting are limited in their applicability to predict outcomes of patients who underwent BITA grafting.

In the current study, we tried to develop a model that identifies predictors for long term survival, up to fifteen years after BITA grafting and use it as a simple validated prediction tool. This model may provide a longer prediction period than the previous ones [7–15], and also specifically addresses the sub group of patients treated with BITA, who were not included in previous studies.

In the current study, we divided all BITA patients operated in our department between 1966 and 2011 into two groups: One thousand four hundred and sixty-eight were the learning group and 1,467 were the validation group. The two groups had similar overall mortality and similar 5, 10, and 15-year survival.

Older age, DM, COPD, CHF, CRF, old MI, EF≤30%, pre-operative intra-aortic balloon, and PVD, were significant predictors for mortality in the current model. A previous model proposed by Wu et al. [7] for prediction of 7-year mortality after CABG included age, body mass index (BMI), EF, hemodynamic stability, left main coronary artery disease, CVD, PVD, CHF, malignant ventricular arrhythmia, COPD, DM, CRF, and previous CABG. Another previous model proposed by MacKenzie et al. [14] for prediction of 8-year mortality after CABG included age, BMI, COPD, DM, EF, gender, left main coronary artery disease, old MI, number of diseased vessels, prior CABG, PVD, CRF, patients' status (elective/urgent, salvage, emergent), and white blood cells. Patients with malignant ventricular arrhythmia were not referred to BITA in our center and therefore this risk factor was not included in our model. Additionally, data on patients' BMI was not available for a large proportion of the charts, and was therefore waived. However, most of the variables that were included in these previous studies were also included as potential predictors in our study.

The Harrell's C in the current model was close to that reported by MacKenzie et al [14]. The AUCs of the cox model for 5, 10, and 15-year survival for the learning group ranged between 0.742 and 0.762, and for the validation group between 0.766–0.770, respectively. The AUCs value for 5 years were close to that reported in previous studies [7,14].

This study has several limitations. First, in order to evaluate long term outcome, we had to use data on patients who underwent CABG between 1996 and 2011. There was an improvement in patient management which may only be partially reflected in the cohort. Second, due to the historical nature of the study, data on BMI and blood tests were available only in part of the patients' charts, and therefore these variables were not included as potential predictors. Third, we did not have data on specific cause of death and therefore all-cause mortality was used instead. Lastly, supplemental angiographic data that could have been included in the model was not available.

In conclusion, a simple to use validated model can be used to predict 5, 10, and 15-year mortality after CABG using the BITA grafting technique.

## Supporting information

S1 Fig ROC curves of the Cox model for 5, 10, and 15-year survival in the learning group. (TIF)

S2 Fig ROC curves of the Cox model for 5, 10, and 15-year survival in the validation group. (TIF)

## Abbreviations

CABG: Coronary artery bypass grafting
LITA: Left internal thoracic artery
LAD: Left anterior descending
SVG: Saphenous vein graft
BITA: Bilateral internal thoracic arteries
SITA: Single internal thoracic artery
DM: Diabetes mellitus
COPD: Chronic obstructive lung disease
CRF: Chronic renal failure
CVD: Cerebral-vascular disease
PVD: Peripheral vascular disease
ND: Neurologic dysfunction
CHF: Congestive heart failure
MI: Myocardial infarction
EF: Ejection fraction
PCI: Percutaneous transluminal coronary angioplasty
LM: Left main
IABP: Intra-aortic balloon
SD: Standard deviation
BMI: Body mass index
