# Supplementary figures and images for: A simple-to-use nomogram to predict long term survival of patients undergoing coronary artery bypass grafting (CABG) using bilateral internal thoracic artery grafting technique
Source: PLoS One. 2019 Oct 24;14(10):e0224310. doi: 10.1371/journal.pone.0224310 (PMC6812830; doi:10.1371/journal.pone.0224310)

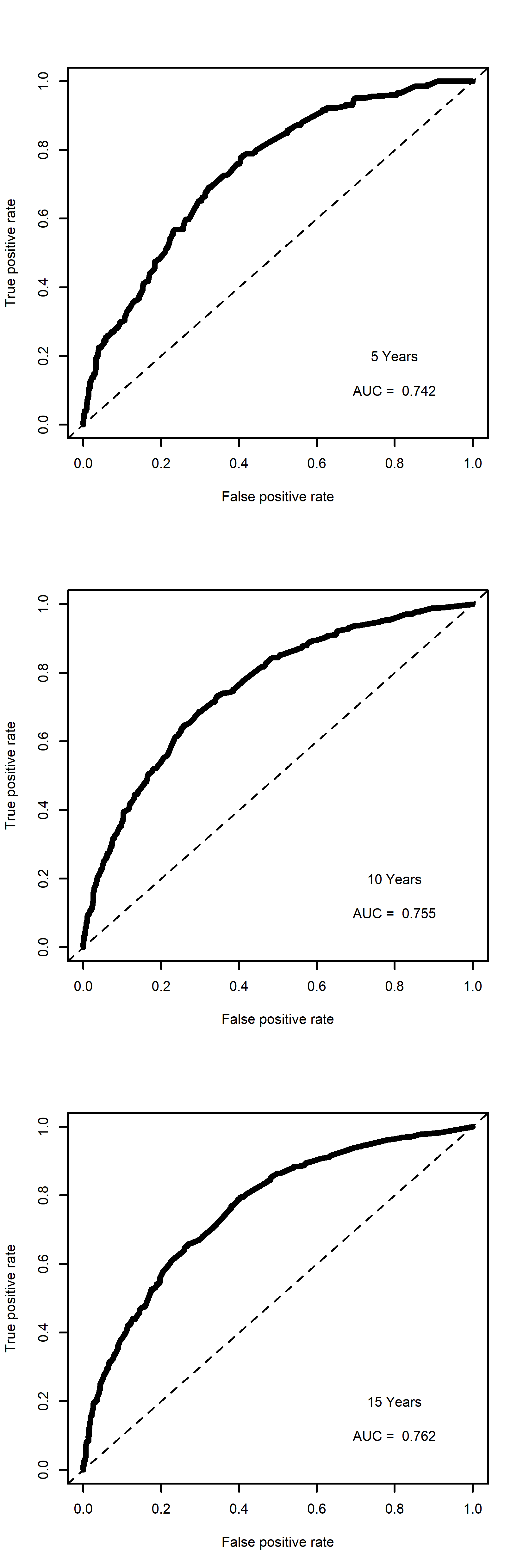

Supplement: S1 Fig — (TIF) [file pone.0224310.s001.tif]

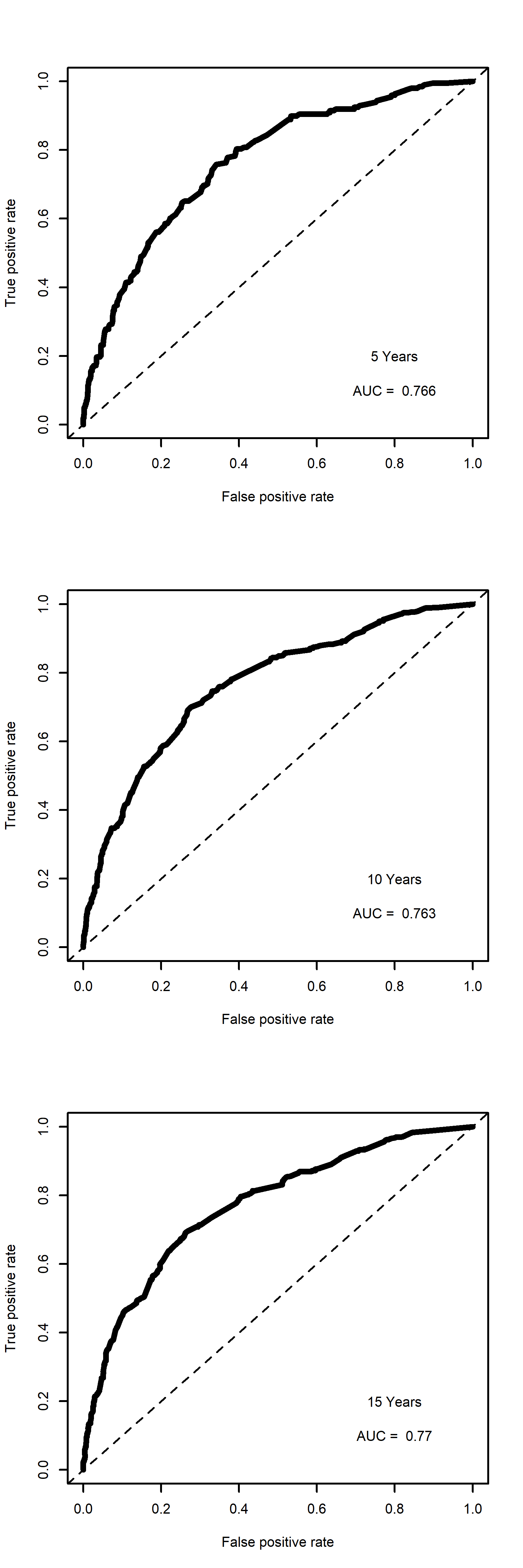

Supplement: S2 Fig — (TIF) [file pone.0224310.s002.tif]
